# Numerical Analysis on Mechanical Properties of Different Fiber-Reinforced Cold-Formed Steel–Concrete Composite Corner Columns

**DOI:** 10.3390/polym17172365

**Published:** 2025-08-30

**Authors:** Mengyao Li, Yi Hu, Lanzhe Rao, Liqiang Jiang, Jingbin Li, Shizhong Zhou, Hongyu Sun, Shi Peng, Xia Pang, Yuanjun Chen, Jun Hu, Ping Xie

**Affiliations:** 1 School of Civil Engineering, Central South University of Forestry and Technology, Changsha 410004, China; 20241200668@csuft.edu.cn (M.L.); huyi@csu.edu.cn (Y.H.); 20211200551@csuft.edu.cn (H.S.); 2 Sichuan Provincial Engineering and Technology Research Center for Innovative Development of Bamboo Fiber Nutrition, Leshan 614000, China; 3 3rd Construction Co., Ltd. of China Construction 5th Engineering Bureau, Changsha 410004, China; 3508731642@126.com (L.R.); 1522954324@126.com (S.P.); 857668751@126.com (X.P.); 4 School of Civil Engineering, Central South University, Changsha 410075, China; 15034738691@163.com; 5 China Railway Urban Construction Group Co., Ltd., Changsha 410036, China; 18669034208@163.com (J.L.); chenyuanjun_csu@foxmail.com (Y.C.); 13787137781@163.com (J.H.); 17788979120@163.com (P.X.)

**Keywords:** cold-formed steel, fiber-reinforced concrete, composite corner column, numerical analysis, ultimate capacity calculation

## Abstract

To overcome brittle failure in conventional cold-formed steel–concrete (CFS-C) corner columns, this paper used fiber-reinforced concrete to replace ordinary concrete, investigating failure mechanisms and performance through systematic numerical simulations. A finite element model (FEM) was established and validated by experiments, and the errors for ultimate capacity were within 10%. A series of numerical models was established for parametric analyses focusing on the effects of the parameters of polypropylene fiber (PF), carbon fiber (CF), steel fiber (SF), and bamboo fiber (BF) with different volume dosages and the thickness of cold-formed steel (CFS) on the axial compression ultimate capacity and corresponding displacement of CFS composite corner columns. The results indicated that (1) PF effectiveness was dependent on steel thickness: thicker steel suppressed micro-defects, activated the toughening potential of PF, and increased the ultimate capacity of the columns by 24.8%. (2) CF had a critical dosage of 0.4%: at this dosage, CF increased the column’s ultimate capacity by 14.1% through stress redistribution, while when the dosage exceeded this value, fiber agglomeration caused a reduction in the column’s strength, with a maximum decrease of 16.2%. (3) SF effectiveness showed a linear increase: at a dosage of 1.6%, SF formed a synergistic three-dimensional bridging network and generated a confinement effect, increasing the column’s ultimate capacity by 36.5% and displacement by 92.2%. (4) BF mainly improved the ductility of columns: through crack bridging and pull-out energy dissipation, BF increased column displacement by 33.2%. (5) The modified Eurocode 4 formula could reduce the calculation error of ultimate capacity from 6.3% to within 1%. The findings guide optimal fiber selection and dosage in practice, promoting such columns’ use in seismic and load-bearing structures.

## 1. Introduction

Cold-formed steel–concrete (CFS-C) composite structures have been increasingly and widely adopted in modern building structures due to their advantages of light self-weight, convenient construction, and high bearing capacity [1,2,3,4]. As an important form of special-shaped column, composite corner columns can effectively meet the spatial requirements of building corners, optimize structural layouts, and improve the service efficiency of buildings [5,6,7,8,9]. However, the mechanical properties of composite corner column members, especially their axial compression capacity and deformation characteristics, are complexly affected by the wall thickness of cold-formed steel (CFS), the performance of core concrete, and the synergistic working mechanism between the two [10,11,12]. In particular, the brittle failure mode of core concrete often becomes a critical factor that limits the overall ductility and load-bearing potential of the members. Fiber-reinforced concrete has been shown to significantly improve concrete’s toughness, crack resistance, and energy absorption capacity [13,14,15,16,17]. However, a comprehensive theoretical system and efficient design methods have not yet been established regarding the systematic influence laws of different types of fibers and their volume dosages on the mechanical properties of CFS-C composite corner columns, as well as the accurate prediction of the axial compression capacity of such fiber-reinforced composite columns. This has greatly restricted the optimal application of high-performance fiber materials in composite corner column structures, and in-depth research is urgently needed to provide reliable theoretical support and a design basis.

In the last few years, many researchers have carried out studies on CFS tube concrete columns, laying a foundation for understanding their basic mechanical behaviors [18,19,20,21]. Chen et al. [22] carried out axial compression experiments on 14 square concrete-filled steel tube column specimens with cross-sectional widths between 156 mm and 1040 mm, investigating the influence of cross-sectional width and steel content on their size effect behavior. The findings showed that the confinement of steel tubes weakened the size effect of peak axial compressive stress. The peak strain, residual strain corresponding to 0.85 times the ultimate capacity, and ductility coefficient all exhibited a downward trend when the section width grew. Li et al. [23] studied the axial compression behavior of wide rectangular concrete-filled steel tube columns (WR-CFST) through axial compression tests and finite element parameter analysis. The results indicated that all WR-CFST underwent weak-axis bending failure, and an increase in the cross-sectional width-to-thickness ratio weakened the confinement effect in the middle and high regions and reduced ductility. The Mander confinement model [24] was applicable to the bearing capacity prediction of specimens with a width-to-thickness ratio of 2–4. Existing codes mostly overestimated the stability coefficient, based on which a simplified prediction formula for the stability coefficient was proposed. Liu et al. [25] investigated the axial compression behavior of short columns with stainless steel tubes filled with circular steel-reinforced concrete through axial compression tests and finite element simulations on six specimens. The findings demonstrated that these short columns had excellent bearing capacity and residual bearing capacity. The steel skeleton inhibited the propagation of diagonal cracks in concrete and improved ductility, and the combination of low-strength concrete with a steel skeleton can enhance the residual bearing capacity. These studies also confirm that establishing refined numerical models using finite element software such as ABAQUS is an effective approach to analyzing the performance of complex composite members. With reasonable material constitutive relations, interface models, and failure criteria, the entire loading process of the members can be simulated relatively accurately [26,27,28].

In the field of fiber-reinforced concrete, research on ordinary rectangular or square columns reinforced with a single type of fiber has made some progress, revealing the positive role of fibers in improving the confining effect of concrete and delaying the local buckling of steel components [29,30,31]. Liu et al. [32] conducted axial compression tests on 36 specimens with different concrete strength grades, steel tube thicknesses, and SF volume dosages, and established finite element models (FEMs) to investigate the effect of SFs on the mechanical properties of concrete-filled square steel tube short columns. The results indicated that an increase in SF volume dosage led to a slight growth in the ultimate capacity of specimens, and a significant improvement in energy dissipation capacity and ductility. However, a volume dosage exceeding 1.2% would have a negative impact. Moderate SFs could delay the failure of core concrete and increase its ultimate load, and a corresponding calculation equation for ultimate load was presented. Zong et al. [33] prepared 25 long column specimens of square steel tubes filled with SF-reinforced recycled coarse aggregate concrete, investigating the effect of the recycled coarse aggregate substitution ratio, tube wall thickness, length–width ratio, and SF dosage on their axial compression performance. The findings demonstrated that the length–width ratio and tube wall thickness significantly affected the mechanical behavior through the confinement effect and instability. When the SF volume dosage was ≥1.2%, the post-peak performance could be improved by anchoring and bridging, compensating for the bearing capacity loss caused by parameter changes. After comparing with code models, an incremental iteration model was proposed, and the predicted values were in good agreement with the test results.

Codes such as Eurocode 4 [34] provide a theoretical framework for calculating the bearing capacity of ordinary concrete-filled steel tube columns [35,36,37,38]. However, when many scholars study the ultimate capacity of special-shaped columns, the results calculated by theoretical formulas have large errors. Wang et al. [39] calculated and analyzed the ultimate capacity of T-shaped CFS composite columns using codes from China, the United States, Britain, Japan, Europe, and other countries to study the applicability of different codes. The results indicated that the calculation errors of GB50018-2002 [40] and AISI S100 [41] codes for hollow columns were within 15%, with good effects. However, the existing codes had large prediction errors for foam concrete-filled columns, which were difficult to calculate accurately due to the complex special cross-sections and composite effects. Liu et al. [11] investigated the prediction effectiveness of different methods for the axial compressive capacity by comparing the experimental capacity of short special-shaped concrete-filled steel tube columns with the design values from existing codes and the calculated values from the methods proposed by them. The results indicated that existing codes either underestimated or overestimated the bearing capacity, while their proposed methods exhibited better prediction accuracy. With accuracy between Eurocode 4 [34] and GB 50936-2014 [42], these methods were relatively reasonable. Furthermore, when these formulas are directly applied to fiber-reinforced CFS-C composite corner columns, the calculation accuracy is often insufficient, as they fail to fully account for the differential contributions of different fiber types and their volume dosages to the overall performance of the composite columns.

Of particular note is that there are three key gaps in current research. Firstly, the mechanism of action of bamboo fibers (BFs), as an emerging environmentally friendly reinforcing material, in composite structures remains unclear. Secondly, the existing literature has not systematically revealed the synergistic reinforcement laws between steel thickness and different fiber-reinforced concretes in corner columns, making it impossible to accurately predict the differential impact of fiber content on the bearing capacity–displacement relationship. Most importantly, existing specifications fail to integrate fiber reinforcement mechanisms with the characteristics of special-shaped cross-sections, resulting in poor accuracy in predicting the bearing capacity of fiber-reinforced corner composite columns. These gaps have severely restricted the engineering application of high-performance fibers in special-shaped columns.

Therefore, this study aims to construct an analytical model of CFS-C corner composite columns reinforced by different fibers through a refined numerical model verified by experiments. It focuses on three core objectives: clarifying the regulatory mechanism of steel thickness and fiber type in the synergistic reinforcement effect; quantifying the differential influence law of the volume dosages of polypropylene fibers (PFs), carbon fibers (CFs), steel fibers (SFs), and BFs on the bearing capacity–displacement relationship; and proposing a modified formula for axial compressive bearing capacity integrating fiber reinforcement characteristics to fill the theoretical gaps in existing specifications, thereby providing a reliable scientific basis for the design of high-performance special-shaped composite columns.

## 2. Materials and Methodology

### 2.1. Component Creation and Assembly

**Figure 1:**
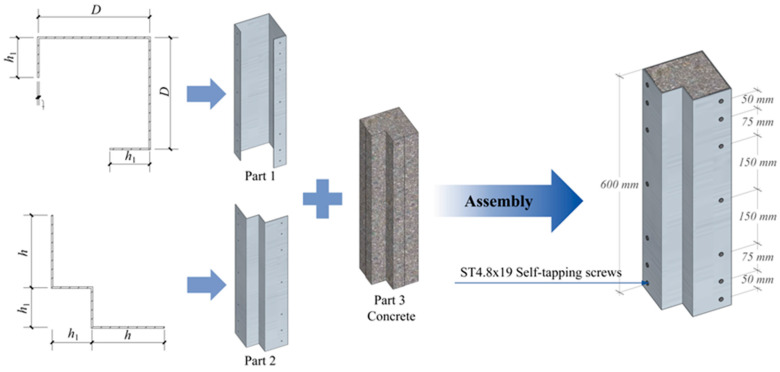
Assembly of the composite column.

The CFS-C composite corner column was mainly composed of two CFS components and concrete, which were connected as a whole by ST4.8 × 19 self-tapping screws, as illustrated in Figure 1. The height of the composite column was 600 mm, and the specific dimensions of the cross-section are presented in Table 1. In ABAQUS/CAE 2022 software, the S4R shell element in the Part module was selected for simulating the steel composite components of the column; the C3D8R solid element was selected for modeling the concrete component, self-tapping screws, and prefabricated end plates. All components were modeled and assembled according to the actually measured dimensions. A three-dimensional model was created through the extrusion command. The hollow extrusion command was used to create holes at the corresponding positions in the steel components, and holes were retained at other corresponding positions.

**Figure 2:**
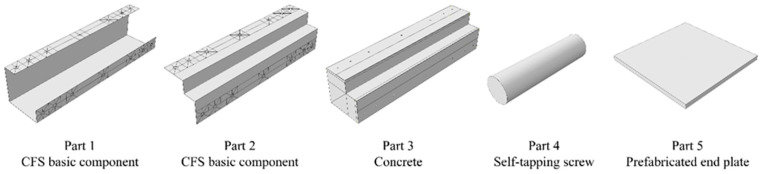
Component modeling.

In the Assembly module, commands such as translation, rotation, and array were used to connect and assemble each component. Considering that the hole-drilling operation might cause problems in mesh division for the simulated specimens, the mesh near the hole positions on the steel components was finely divided; the solid module on the concrete component was split to ensure that the mesh division and simulation calculation of the components were not affected by the hole-drilling operation. The modeling of each component is shown in Figure 2.

### 2.2. Material Properties

#### 2.2.1. Steel

**Figure 3:**
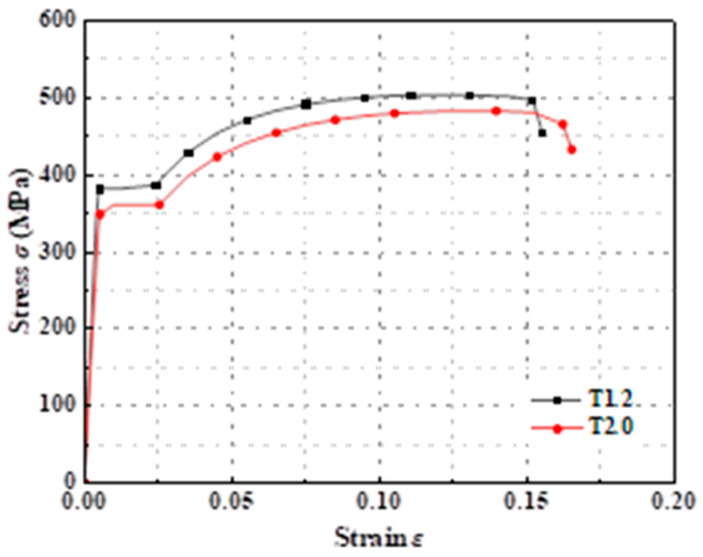
Stress–strain relationship curve of steel.

The model used Q345 CFS with 1.2 mm and 2.0 mm thickness. The stress–strain values of the steel gained in previous research of the research group were integrated into the FEM for simulating the nonlinear characteristics of the steel [39]. The Young’s modulus of the specimens was 2.06 × 10^5^ Mpa, and the Poisson’s ratio was 0.3. The stress–strain relationship curve of steel is shown in Figure 3 [39]. The specimen numbers “T1.2” and “T2.0” indicate that the thicknesses of the steel materials are 1.2 mm and 2.0 mm, respectively. The yield strengths of the 1.2 mm and 2.0 mm thick specimens were 503.3 Mpa and 482.3 Mpa, respectively. In the test results of the composite column axial compression test, there was no evidence of screw failure in terms of shear, tilting, or pull-out. This indicated that the stiffness of the screws was sufficient to prevent sliding between the overlapping sections of each basic component. Therefore, the yield stress of the self-tapping screws was set to 400 Mpa, the plastic strain was set to 0, and the Young’s modulus and Poisson’s ratio were consistent with those of the test specimens.

#### 2.2.2. Core Concrete

The material properties of concrete also adopted the concrete stress–strain values obtained from the experiments of the research group. The concrete damage plasticity (CDP) model was used, which could well simulate the nonlinear behavior of concrete under monotonic loading. In this study, the uniaxial constitutive relationship of concrete proposed by Huang et al. [29] was adopted to characterize the stress–strain behavior of concrete. The key CDP parameters used in the model are presented in Table 2. The Young’s modulus of the two concretes with different mix proportions was 11,691 Mpa and 14,987 Mpa, respectively, and both had a Poisson’s ratio of 0.2.

### 2.3. Constraints and Loads

The model was loaded using an explicit setting. This was because the connection between concrete and steel in the composite column was relatively complex, making it difficult to achieve proper assembly. During the loading process of composite columns using static settings, phenomena such as component interpenetration due to assembly errors or calculation non-convergence often occurred. However, the use of explicit calculation could perfectly solve this series of problems, while shortening the calculation time and reducing the waste of computing power.

**Figure 4:**
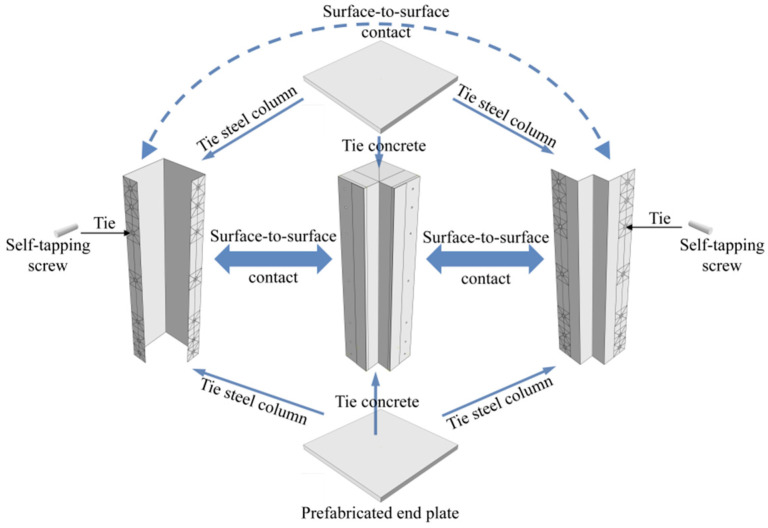
Interaction settings of the model.

In the Interaction module, surface-to-surface contact was adopted between the composite steel components, with the contact properties set to tangential frictionless and normal hard contact, ensuring that the surface-to-surface contact could only transmit normal pressure without mutual penetration. Surface-to-surface contact was also adopted between the composite steel components and concrete, with the contact properties set to tangential penalty behavior (with a friction coefficient of 0.3) and normal hard contact. The coefficient of friction was determined by referring to the research of Wilches et al. [43]. The penalty behavior could well simulate the contact and interaction between steel and concrete. The contact between the prefabricated end plates and the composite column, and between the screws and the composite column, was bound using the tie command. The interactions between components are shown in Figure 4.

**Figure 5:**
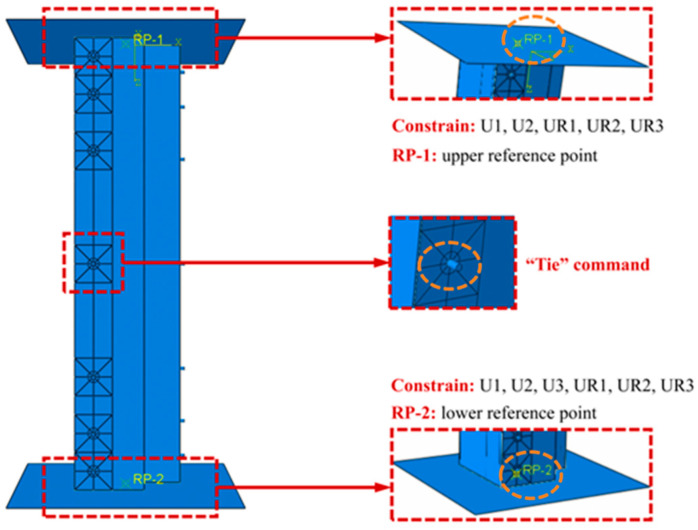
Assembled model.

During the test loading process, the specimen was directly placed on the instrument pedestal, with the center point of the specimen aligned with that of the pedestal, and displacement loading was conducted by moving the lower pedestal. Therefore, in the FEM, to guarantee consistency with the actual loading conditions of the test, reference points (RF-1 and RF-2) were placed at the centers of the prefabricated end plates on the two ends. To simulate the actual boundary constraints in the axial compression test, all degrees of freedom of RF-1 except the U3 direction were restricted; RF-2 was subjected to a fixed-end constraint, and axial displacement was applied through the U3 direction of RF-1 to simulate displacement loading. Details are shown in Figure 5.

### 2.4. Mesh Division

**Figure 6:**
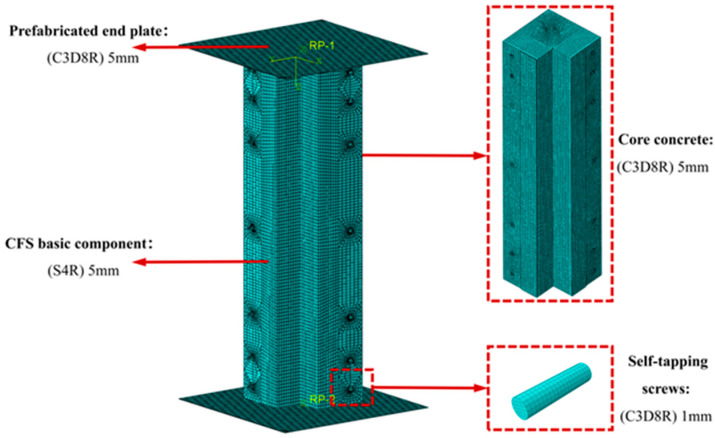
Meshing of components.

In this study, the model was meshed using a structurally optimized mesh division method. Due to the presence of numerous perforated regions in the composite column components, irregular mesh patterns might occur near these openings during the meshing process. Therefore, the corresponding perforated regions were split before mesh division. The CFS basic components were modeled using quadrilateral shell elements (S4R) with a uniform seed size of 5 mm, containing a total of 14,355 nodes and 13,758 elements. The prefabricated end plates used swept hexahedral elements (C3D8R) as the base units, also with a seed size of 5 mm, generating 3721 nodes and 3600 elements. Self-tapping screws were modeled using swept hexahedral elements (C3D8R) refined to 1 mm, including 820 nodes and 608 elements. The concrete component in this study had an irregular cross-sectional shape, and holes were drilled on its surface to simulate the actual assembly. These holes could lead to poor mesh quality during the meshing process of the concrete component; therefore, the entire concrete component needed to be split. Concrete components adopted swept hexahedral elements (C3D8R) with a seed size still set to 5 mm, eventually forming 132,045 nodes and 119,282 elements. The meshing of the components is shown in Figure 6.

## 3. Results and Discussion

### 3.1. Validation of the FEM

#### 3.1.1. Comparison of Failure Phenomena

**Figure 7:**
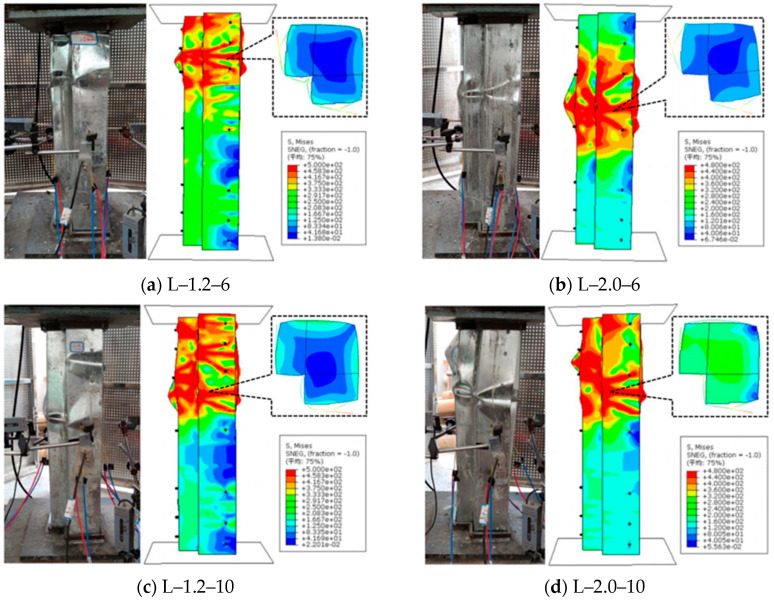
Comparison of specimen failure modes.

The test data of the specimens L-1.2-6, L-2.0-6, L-1.2-10, and L-2.0-10 were obtained from previous research of the authors’ team. As shown in Figure 7, the loading results of the FEM are roughly consistent with the failure phenomena and positions observed in the actual loading process of the test specimens. This demonstrates that the modeling approach adopted in this study is relatively effective and reliable for the numerical analysis of concrete composite column specimens. However, there are certain deviations in the failure positions and phenomena between some finite element simulation specimens and the actual test results. This is because the loading of the finite element specimens consists of an axial compression test under ideal conditions, where there is no deviation during the assembly of the specimen and in the alignment between the specimen and the lower pedestal of the loading instrument. Meanwhile, the simulated specimens do not have the problems of sedimentation of dry concrete grouting materials at the bottom and uneven concrete at the ends.

#### 3.1.2. Comparison of Load–Displacement Curves

**Figure 8:**
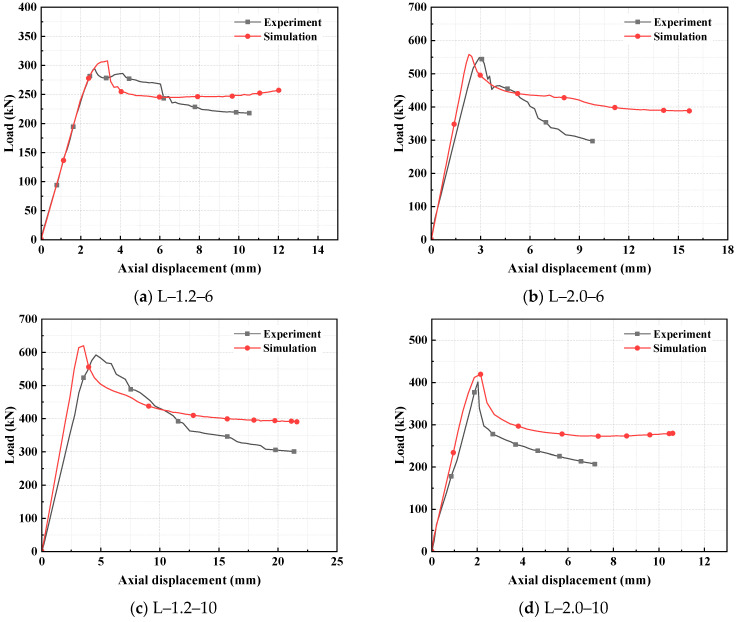
Comparison of load–displacement curves of specimens.

Figure 8 shows that there is a good agreement between the load–displacement curves obtained from experiments and those from finite element simulations, especially in terms of the prediction of initial elastic stiffness and peak load. Meanwhile, since the simulated specimens do not account for actual errors and their cross-sections adopt a spliced configuration, the stiffness of the simulated specimens is relatively higher. This leads to a certain deviation between the simulation results and the curves of the experimental specimens, but the overall variation trend remains consistent. The main discrepancy occurs in the post-peak phase: due to the gradual physical damage to materials and the inherent variability in experiments, the experimental curves exhibit obvious fluctuations compared with the simulation curves; the simulation curves of specimens L-1.2-6 and L-2.0-10 show a slight recovery after a decline. This numerical hardening is attributed to contact re-stabilization and internal stress redistribution within the ABAQUS model, where re-engagement of contact surfaces and constrained localization after initial softening can temporarily restore stiffness. Although this specific fluctuation is a numerical artifact of the explicit solver managing extreme material nonlinearity and geometric instability, the overall correlation in the pre-peak response, peak load, and global trend validates the model’s effectiveness for parametric analysis.

#### 3.1.3. Comparison of Ultimate Capacity

As can be observed in Table 3, the specimen finite element and test errors are within 10%, which proves the accuracy and viability of the finite element modeling approach adopted in this study.

### 3.2. Parameter Analysis of CFS-C Composite Corner Columns

In this chapter, the validated FEM in the previous section was used to conduct a parametric study, investigating the effects of four types of fiber-reinforced concrete (PF, CF, SF, and BF) and four steel plate thicknesses (*t* = 1.2 mm, 1.5 mm, 1.8 mm, and 2.0 mm) on the mechanical properties of CFS-C composite corner columns. The basic properties of the four types of fiber materials are presented in Table 4. A total of 72 specimen models were established.

#### 3.2.1. Polypropylene Fiber-Reinforced Concrete

The material property test data of PF-reinforced concrete were derived from reference [44]. The study investigated the influence of PF volume dosages (*V*_f_) of 0%, 0.5%, 1.0%, 1.5%, and 2.0% on the ultimate capacity of CFS-C composite corner columns. The specimen numbering rule was “PF-1.2-0”, where “PF” represented polypropylene fiber, “CF” represented carbon fiber, “SF” represented steel fiber, and “BF” represented bamboo fiber; “1.2” denoted the steel thickness in millimeters; and “0” indicated the volume dosage of PFs in the concrete.

**Figure 9:**
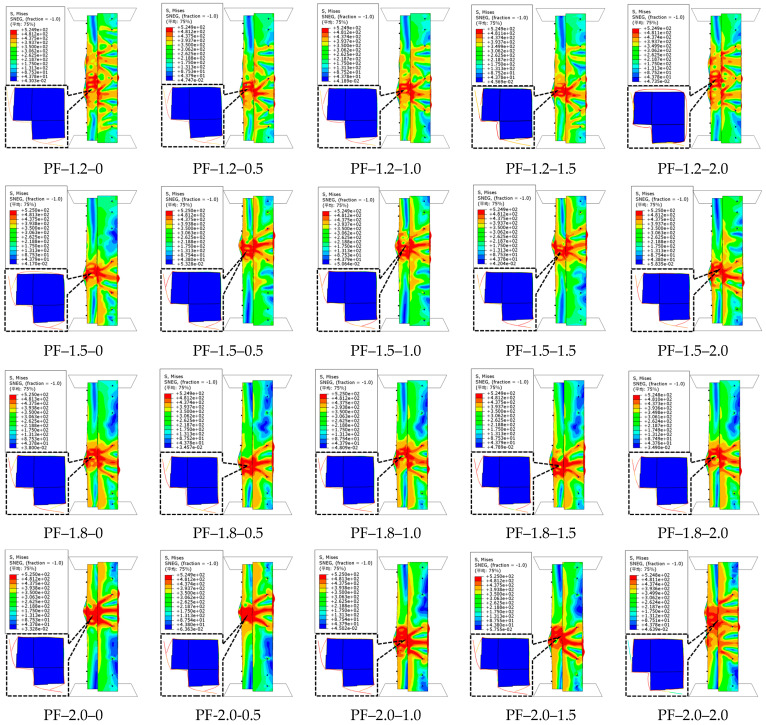
Failure characteristics of CFS–PF–reinforced concrete composite columns.

**Figure 10:**
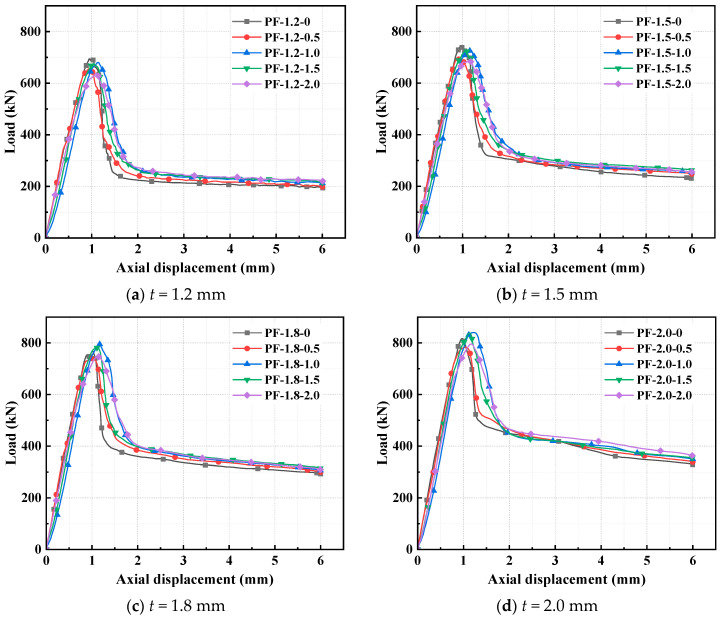
Load–displacement curves of CFS-PF-reinforced concrete composite columns.

The failure characteristics of the CFS-PF-reinforced concrete column specimens are illustrated in Figure 9. The failure mode of the specimens is mainly local buckling, occurring at the central part with bulging on the short side of the corner column. Figure 10 indicates the relationship between specimen load and displacement. For specimens with steel thicknesses *t* of 1.2 mm and 1.5 mm, when the volume dosage of PFs is 2.0%, the maximum reduction in the ultimate capacity of the specimens is 8.3% and 11.8%, respectively. When the volume dosage is 1.0%, the displacement at ultimate capacity increases the most, by 8.1% and 16.7%, respectively. The ultimate capacity shows an overall decreasing trend, mainly because the incorporation of PFs disrupts the uniformity of the cement paste, leading to a small decrease in the compressive strength of concrete. When the volume dosage of PFs is 1%, the specimens with steel thicknesses of 1.8 mm and 2.0 mm showed the largest increases in ultimate capacity, 3.5% and 2.9%, respectively; meanwhile, the corresponding displacements showed the largest increases, 16.2% and 24.8%, respectively. This indicates that steel with *t* ≥ 1.8 mm inhibits the development of microcracks and pore defects in concrete caused by PFs, thereby improving the ultimate capacity. During the axial compression of the specimen, the bonding, slipping, and pulling out between PFs and the concrete matrix absorb a large amount of energy, which enhances the deformation capacity and fracture energy of concrete, and further improves the ductility of the specimen. This trend of ultimate capacity varying with steel thickness reveals the synergistic critical value between CFS confinement and fiber action. When the steel thickness is insufficient, matrix defects caused by fiber dispersion dominate; once the thickness reaches the required standard, the triaxial stress field generated by confinement can activate the energy-dissipating advantage of fibers. This critical transition mechanism provides a quantitative basis for parameter matching between fibers and steel. When the steel thickness increases from 1.2 mm to 2.0 mm, the ultimate capacities of the specimens with five PF volume dosages increase by 17.4%, 19.4%, 23.4%, 24%, and 24.8%, respectively. This indicates that the steel thickness has a significant influence on the ultimate capacity of the specimens.

#### 3.2.2. Carbon Fiber-Reinforced Concrete

The material property test data of CF-reinforced concrete were derived from reference [45]. This study investigated the influence of CF volume dosages (*V*_f_) of 0%, 0.2%, 0.4%, 0.6%, and 0.8% on the ultimate capacity of CFS-C composite corner columns.

**Figure 11:**
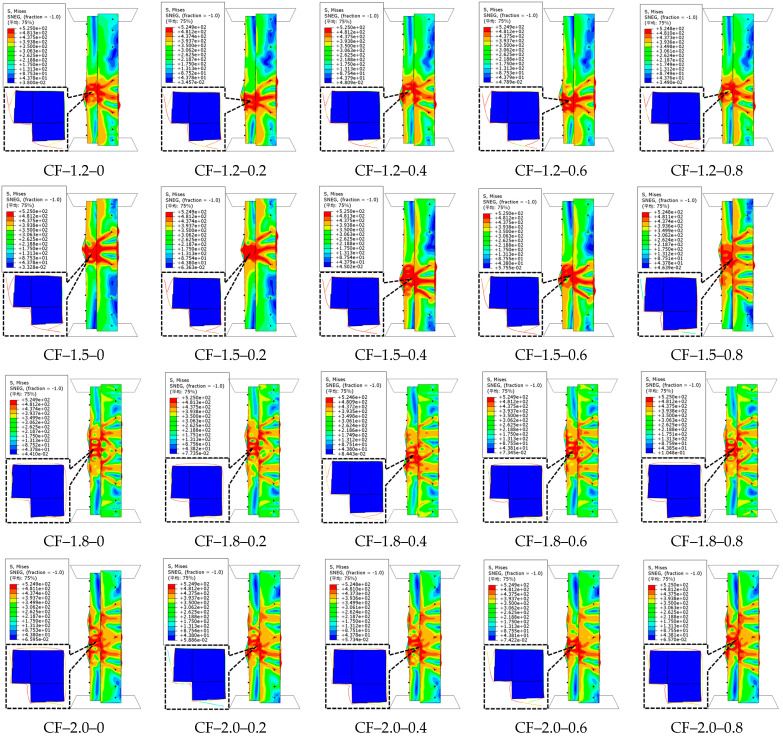
Failure characteristics of CFS–CF-reinforced concrete composite columns.

**Figure 12:**
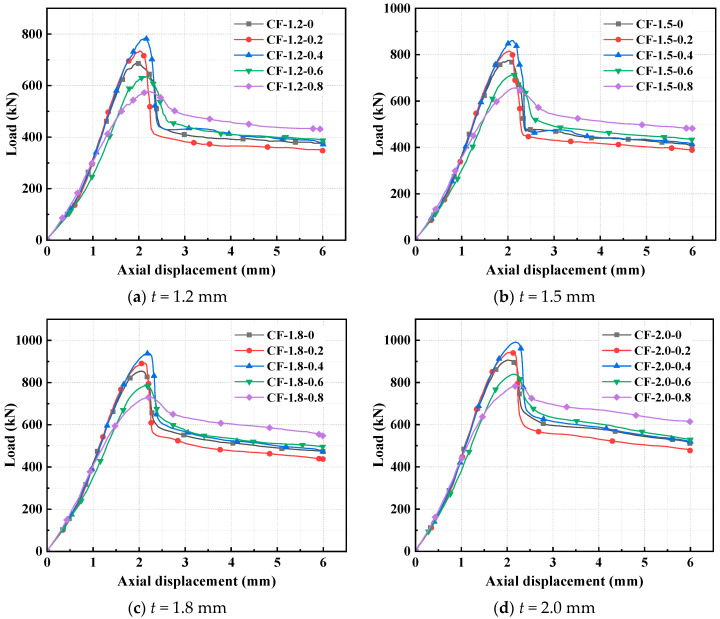
Load–displacement curves of CFS-CF-reinforced concrete composite columns.

The failure characteristics of the CFS-CF-reinforced concrete column specimens are illustrated in Figure 11. The failure mode of the specimens is similar to that of the CFS-PF-reinforced concrete columns mentioned above. It is observed from Figure 12 that for specimens with the same steel thickness, when the CF volume dosage grows from 0% to 0.4%, the ultimate capacity shows an upward trend; when the CF volume dosage exceeds 0.4%, the ultimate capacity of the specimens starts to decrease and becomes lower than that when the CF volume dosage is 0%. For specimens with steel thicknesses of 1.2 mm, 1.5 mm, 1.8 mm, and 2.0 mm, when the CF volume dosages are 0.2%, 0.4%, 0.6%, and 0.8% respectively, the increase ranges of their ultimate capacities compared to specimens without CFs are approximately as follows: 1.2 mm (6.7%, 14.1%, −7.6%, −16.2%), 1.5 mm (5.3%, 11.2%, −7.8%, −15.3%), 1.8 mm (4.6%, 10%, −8.1%, −14.4%), and 2.0 mm (4.1%, 9.3%, −7.4%, −13.5%). This indicates that when the CF dosage is ≤0.4%, the fibers can be uniformly dispersed in the concrete matrix. Their ultra-high elastic modulus effectively transfers axial stress and inhibits microcrack propagation. Meanwhile, the confinement of the steel tube enables the fibers to give full play to their stress redistribution capacity, thus significantly improving the ultimate capacity. However, when the dosage exceeds 0.4%, the fibers exhibit an agglomeration effect due to a sharp increase in specific surface area, forming local defect zones. This triggers stress concentration and impairs the integrity of the matrix, resulting in a significant decrease in ultimate capacity. As the CF dosage increases, the displacement at ultimate capacity also shows an upward trend. For specimens with the four steel thicknesses, the increases are 3.6~14.7%, 1~6.9%, 3.2~10.2%, and 3.2~12.3%, respectively. This indicates that the increase in CF dosage remarkably enhances the deformation capacity of the specimens. The main reason is that CFs can inhibit concrete crack opening through interfacial bonding force. The stress redistribution effect of fibers delays the accumulation of local damage, transforming the concrete from brittle crushing to progressive failure, thereby improving the ductility of the specimens. The critical dosage of 0.4% not only is a result of this study, but also reflects the dispersion limit of high-modulus fibers in concrete. When the fiber density exceeds the threshold for uniform distribution that the matrix can accommodate, their reinforcing effect will be offset by interface defects. This rule provides a dosage control principle for the engineering application of similar high-modulus fibers [45]. When the steel thickness increases from 1.2 mm to 2.0 mm, the ultimate capacities of the specimens with five CF volume fractions increase by 31.8%, 28.5%, 26.3%, 32%, and 36%, respectively.

#### 3.2.3. Steel Fiber-Reinforced Concrete

The material property test data of SF-reinforced concrete were derived from reference [46]. The influence of SF volume dosages (*V*_f_) of 0%, 1.0%, 1.3%, and 1.6% on the ultimate capacity of CFS-C composite corner columns was studied.

**Figure 13:**
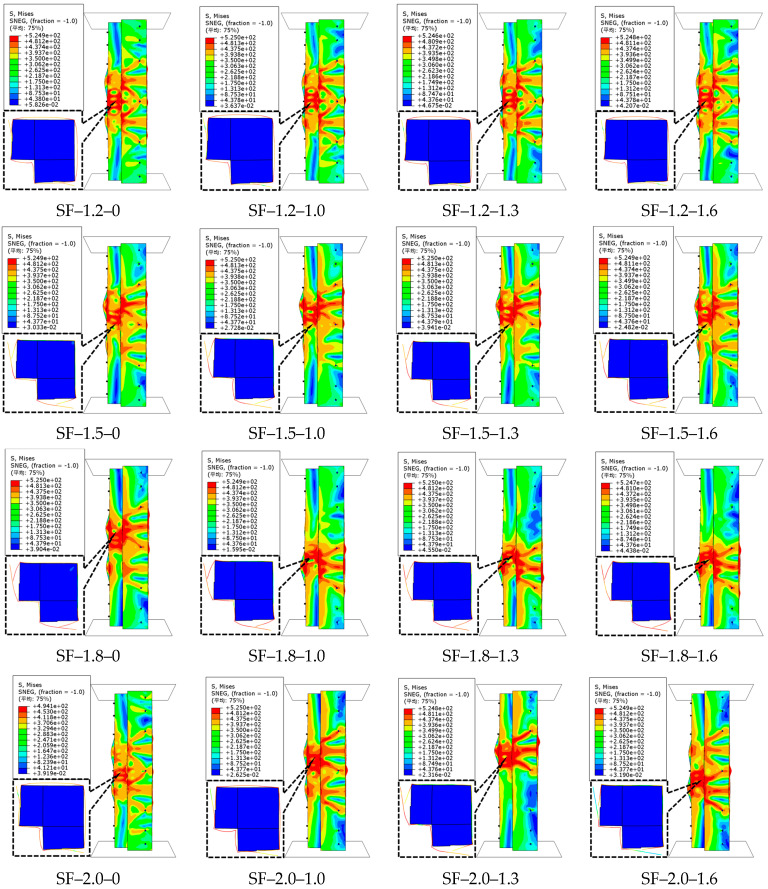
Failure characteristics of CFS–SF–reinforced concrete composite columns.

**Figure 14:**
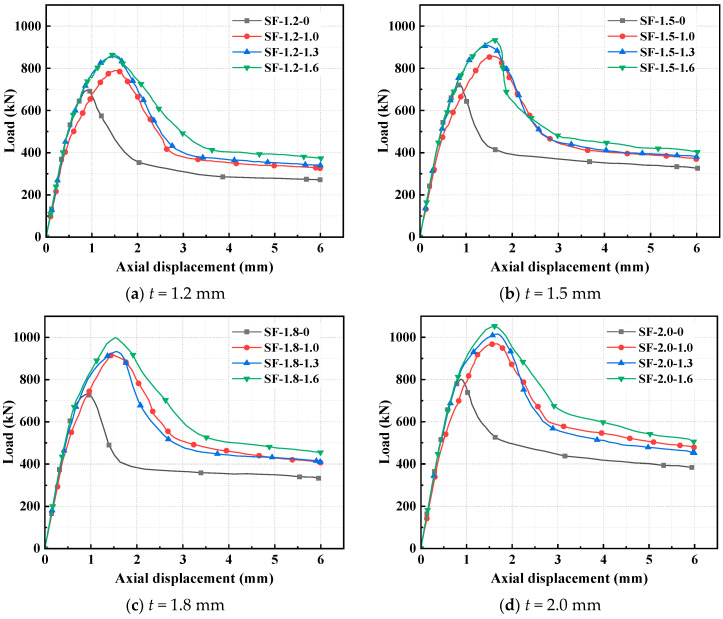
Load–displacement curves of CFS-SF-reinforced concrete composite columns.

The failure characteristics and load–displacement curves of CFS-SF-reinforced concrete composite column specimens are shown in Figure 13 and Figure 14, respectively. When the volume dosage of SFs increases from 1.0% to 1.6%, compared with the specimens without SFs, the ultimate capacities of the specimens with four steel thicknesses increase by 13.4~24.4%, 18.9~29.3%, 25.4~36.5%, and 21~31.4%, respectively. The incorporation of SFs into concrete significantly improves the ultimate capacity of the specimens, mainly because SFs form a three-dimensional bridging network at crack tips. They directly bear tensile stress through their ultra-high tensile strength and inhibit the penetration and propagation of cracks. Meanwhile, SFs and steel tubes produce a synergistic confinement effect. This dual reinforcement mechanism transforms the failure mode of concrete from brittle crushing to progressive failure. As can be evidently observed in Figure 14, the incorporation of SFs significantly increases the displacement at ultimate capacity of the specimens. For the specimens with four steel thicknesses, the displacements corresponding to the ultimate capacity are increased by 62.3~67.4%, 71~89.3%, 60.2~70.9%, and 83.2~92.2%, respectively. The significant increase in displacement stems from the fact that SFs completely alter the energy dissipation mechanism of concrete. The failure energy of ordinary concrete is mainly consumed in the surface energy of cracks, while SFs convert energy dissipation into interfacial friction work and plastic deformation work through the continuous pull-out process, making the load–displacement curve exhibit a gently descending pattern. The linear reinforcement characteristic of SFs is closely related to the stability of their dual mechanism of “three-dimensional bridging-synergistic confinement”. Unlike other fibers that are limited by their dispersibility or modulus, SFs have higher toughness and better compatibility with the concrete matrix, which makes their reinforcement effect increase steadily with dosage [46]. This characteristic endows them with greater application advantages in scenarios requiring high bearing capacity. When the steel thickness increases from 1.2 mm to 2.0 mm, the ultimate capacities of the specimens with four SF volume dosages increase by 15%, 22.7%, 18.8%, and 21.5%, respectively. In summary, the simulation results indicate that there is a positive linear correlation between the volume dosage of SFs and the ultimate capacity. The higher the dosage of SFs, the greater the improvement in the axial compression performance of the specimens.

#### 3.2.4. Bamboo Fiber-Reinforced Concrete

The material property test data of BF-reinforced concrete were derived from reference [47]. The influence of BF volume dosages (*V*_f_) of 0%, 0.25%, 0.5%, and 0.75% on the ultimate capacity of CFS-C composite corner columns was investigated.

**Figure 15:**
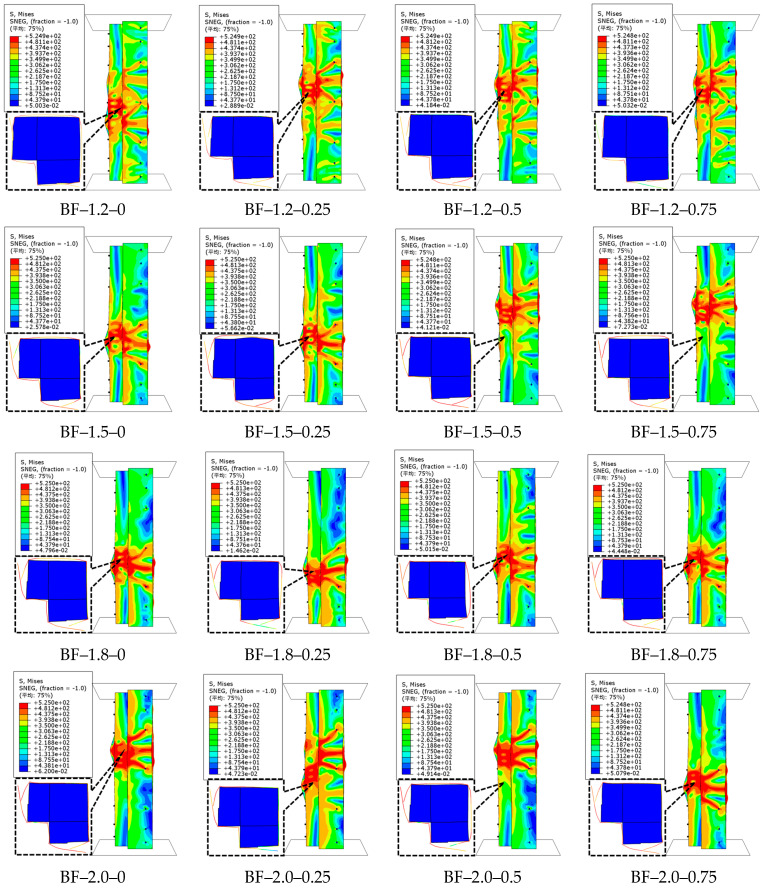
Failure characteristics of CFS-BF-reinforced concrete composite columns.

**Figure 16:**
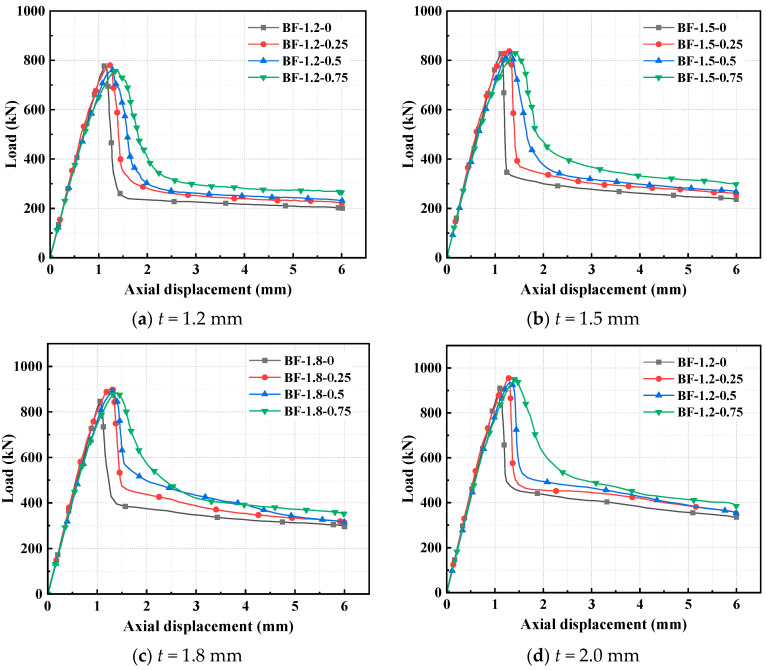
Load–displacement curves of CFS-BF-reinforced concrete composite columns.

The failure characteristics and load–displacement curves of CFS-BF-reinforced concrete composite column specimens are shown in Figure 15 and Figure 16, respectively. For specimens with steel thicknesses *t* of 1.2 mm, 1.5 mm, and 1.8 mm, when the BF volume dosage is 0.25%, the ultimate capacity increases the most, by 0.1%, 2.1%, and 7.4%, respectively. However, when the BF volume dosage is greater than 0.25%, the ultimate capacity shows a downward trend, and even the ultimate capacity of the specimen with a steel thickness of 1.2 mm is lower than that of the specimen without BFs. For the specimen with a steel thickness of 2.0 mm, when the BF dosage is 0.75%, the ultimate capacity increases the most, by 5.3%. This is because BFs have relatively low strength and modulus, and they are hydrophilic. Their incorporation slightly degrades the compactness and compressive strength of the concrete matrix. When *t* ≤ 1.8 mm, the confining stress provided is limited and cannot effectively compensate for the matrix strength loss and the increase in internal micro-defects caused by the increase in BF dosage, so the ultimate capacity declines with the rise in dosage. However, when *t* = 2.0 mm, the strong confining stress generated can significantly inhibit the transverse expansion of concrete, partially offset the matrix deterioration triggered by high BF dosage through the triaxial compression state, and, at the same time, make fuller use of the improved toughness and crack control ability of BFs under confined conditions, thus achieving a small improvement in ultimate capacity at a dosage of 0.75%. Overall, the incorporation of BFs does not significantly improve the ultimate capacity of the specimens, but it remarkably enhances the corresponding displacements.

When the BF dosage increases from 0% to 0.75%, the displacements corresponding to the ultimate capacity of the specimens with four steel thicknesses increase by 20.1%, 26.4%, 33.2%, and 29.6%, respectively. This indicates that the core reinforcement mechanism of BFs lies in their excellent toughening ability. BFs significantly improve the fracture energy and deformation capacity of concrete by bridging microcracks, delaying crack propagation, and gradually being pulled out during the failure process, transforming the failure mode from brittle to more ductile. This toughening effect has relatively low dependence on the thickness of the steel tube and remains effective at high dosages, thus achieving a substantial increase in displacement in specimens with different wall thicknesses. The significant improvement in ductility and limited effect on ultimate capacity by BFs reflect the performance focus of natural fibers. Although their hydrophilic property impairs the compactness of the matrix, their aspect ratio and toughness are more compatible with the energy-dissipating mode of “crack bridging-slow pull–out” [47]. This characteristic of “sacrificing part of the bearing capacity for ductility” endows the fibers with unique value in scenarios with high-deformation requirements such as earthquake resistance. In other words, BFs mainly optimize the ductility and damage tolerance of composite columns rather than their ultimate capacity. When the steel thickness increases from 1.2 mm to 2.0 mm, the displacements corresponding to the ultimate capacity of the specimens with four BF volume dosages increase by 16.8%, 22.4%, 22.7%, and 26.3%, respectively.

## 4. Calculation of Ultimate Capacity Using Eurocode

Based on the finite element parameter variation analysis of different fiber-reinforced CFS-C composite corner columns, and in conjunction with European codes, this study proposed a theoretical calculation formula for the axial compression capacity of composite corner concrete columns, and conducted verification and analysis of its validity and accuracy.

### 4.1. Calculation of Ultimate Capacity

The ultimate capacity of axially compressed specimens was calculated using Eurocode 4 [34]. Eurocode 4 adopts a fully plastic cross-section to superimpose the strengths of various components, and its calculation formula is as follows:

(1) $$N_{u}=\frac{A_{s\;}f_{y}}{\gamma_{s}}+\frac{A_{c\;}f_{c}^{\prime }}{\gamma_{c}}$$

where *N*_u_ is the ultimate axial compression-bearing capacity of the specimen; *A*_s_ and *A*_c_ are the cross–sectional areas of steel and concrete, respectively; *f*_y_ is the yield strength of steel; *f*_c_^’^ is the axial compressive strength of cylindrical concrete specimens; and *γ*_s_ and *γ*_c_ are the partial safety factors for steel and concrete, respectively, with *γ*_s_ = 1.1 and *γ*_c_ = 1.5.

### 4.2. Comparison of Ultimate Capacity Calculations

**Figure 17:**
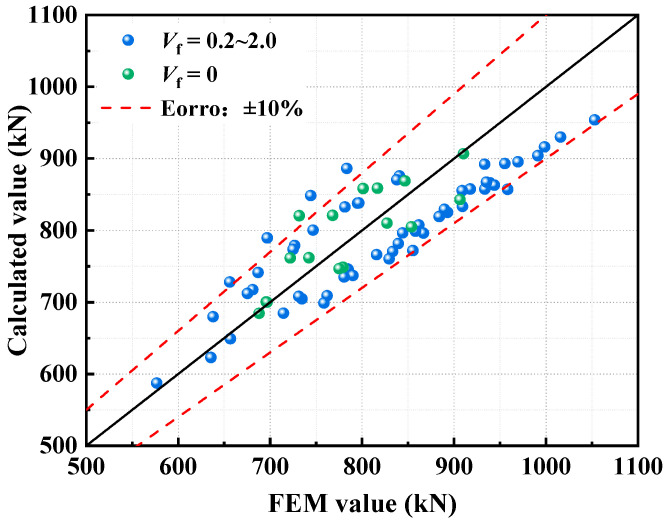
Comparison between simulated values and calculated values.

The comparison between the ultimate capacity values *N*_u_ calculated using Eurocode 4 and the values *P*_f_ derived from finite element simulation analysis is illustrated in Table 5 and Figure 17. The ratio of the calculated values *N*_u_ to the simulated values *P*_f_ ranges from 0.903 to 1.140, with an overall error of 0.063. When no fibers are incorporated, the ratio of the calculated ultimate capacity to the simulated values for the specimens ranges from 0.930 to 1.121, with an overall error of 0.042, which is remarkably lower than that of fiber-reinforced specimens. This indicates that there is a systematic deviation in the bearing capacity prediction of fiber-reinforced CFS-C composite columns using the Eurocode 4 formula. None of the calculated values can fully reflect the enhancement effect of fiber dosage on concrete performance, as Eurocode 4 is based on the strength of plain concrete. Moreover, Eurocode 4 does not consider the change in failure mode caused by fibers. For example, PFs and BFs improve concrete toughness by inhibiting crack development; SFs and CFs enhance crack resistance and bearing capacity by forming a three-dimensional reinforcement network. However, the linear superposition model of the bearing capacities of steel and concrete in the formula neither quantifies the local buckling effect of thin-walled CFS nor includes the fiber–matrix synergistic working mechanism. Meanwhile, the partial safety factors fail to adapt to the strength discreteness of fiber-reinforced concrete, thus leading to errors.

### 4.3. Modification and Verification of the Ultimate Capacity Calculation Formula

The aforementioned analysis indicates that Eurocode 4 is not applicable for calculating the ultimate capacity of fiber-reinforced CFS-C composite corner column specimens. The main issue lies in the enhancement effect of fiber volume dosage on concrete properties. To compensate for this defect, this study proposed a correction factor *η* to modify the Eurocode 4 calculation formula, making it more applicable to the calculation of the ultimate capacity of specimens of fiber-reinforced concrete. The modified formula is as follows:

(2) $$\eta =\frac{P_{f}}{N_{u}}$$

(3) $$N_{u}^{\;’}=\eta N_{u\;}=\eta \;(\frac{A_{s\;}f_{y}}{\gamma_{s}}+\frac{A_{c\;}f_{c}^{\prime }}{\gamma_{c}})$$

**Figure 18:**
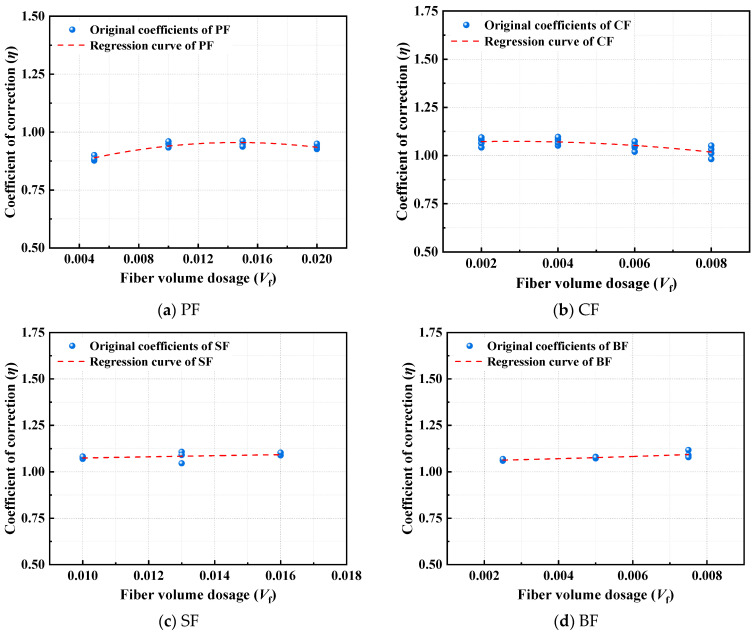
Regression analysis curve.

Based on the analysis of data patterns (Figure 18), an equation relating the fiber volume dosage *V*_f_ to the bearing capacity correction factor *η* is established, as follows:

(4) $$\mathrm{PF}:\;\eta \;=\;-711.15V_{f}^{\;2}\;+\;20.81V_{f}\;+\;0.8$$

(5) $$\mathrm{CF}:\;\eta =-2053.37V_{f}^{\;2}+11.49V_{f}+1.06$$

(6) $$\mathrm{SF}:\;\eta =-73.28V_{f}^{\;2}+4.91V_{f}+1.03$$

(7) $$\mathrm{BF}:\;\eta =241.72V_{f}^{\;2}+3.58V_{f}+1.05$$

**Figure 19:**
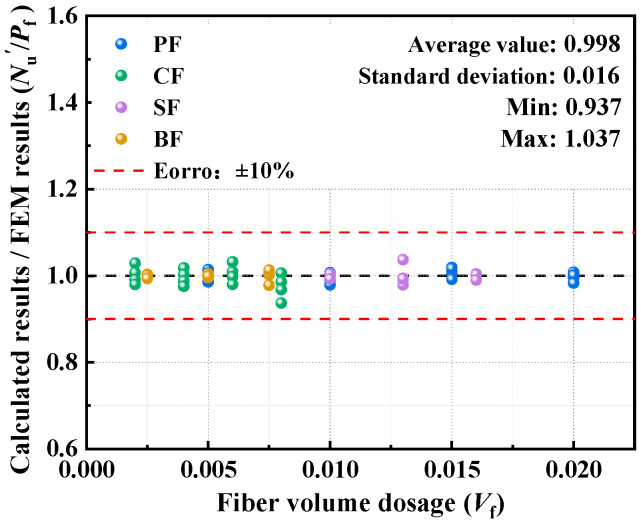
Comparison of modified results.

As shown in Figure 19, after the modification of the Eurocode 4 formula, the ratio of the calculated results to the finite element results ranges from 0.937 to 1.037, with a mean value of 0.998, a standard deviation of 0.016, and an overall error within 1%. This indicates that the improved model, which integrally couples the fiber volume dosage correction factor with the Eurocode 4 formula, effectively unifies the fiber reinforcement effect and the steel–concrete synergistic mechanism, resulting in a significant improvement in prediction accuracy.

## 5. Conclusions

This study developed a refined numerical model for fiber-reinforced CFS-C composite corner columns. Accurate simulations of material nonlinearity, steel–concrete interface effects, and fiber discretization were conducted and validated by four sets of experiments. A systematic parameter analysis was conducted to study the effect mechanisms of four types of fibers. Finally, a modified design method was proposed. The main conclusions are presented below:

(1) The reinforcing effect of PFs showed a significant dependence on steel thickness. When the steel thickness *t* ≤ 1.5 mm, a high dosage of 2.0% led to a decrease in ultimate capacity by up to 11.8%, whereas when *t* ≥ 1.8 mm, a dosage of 1.0% could increase the ultimate capacity by 3.5% and simultaneously increase the corresponding displacement by 24.8%. This phenomenon arose because when the thin-walled steel provided insufficient confinement, fibers induced the propagation of micro-defects in the concrete matrix; in contrast, thicker steel could effectively inhibit crack propagation and activate the potential of fiber bridging for toughening. This indicates that PFs were suitable for components with *t* ≥ 1.8mm, and a dosage of 1.0% can achieve the synergistic optimization of bearing capacity and ductility.
(2) CFs exhibited a clear critical dosage effect. At a dosage of 0.4%, the ultimate capacity was increased by up to 14.1%, but beyond this dosage, performance deteriorated due to fiber agglomeration, with the maximum reduction in bearing capacity reaching 16.2%. It is worth noting that the displacement at ultimate capacity increased continuously with the dosage, with the highest increase reaching 14.7%. The underlying mechanism was that at low dosages, fibers were uniformly dispersed and exerted their high-modulus advantage; however, excessive incorporation formed weak stress concentration zones, while the crack-inhibiting ability of fibers consistently improved ductility.
(3) A comparative analysis of four types of fibers showed that SFs had the most significant effect in simultaneously enhancing the ultimate capacity and ductility of specimens. SFs exhibited a linear enhancement characteristic. When the dosage rose from 1.0% to 1.6%, the ultimate capacity increased by 13.4% to 36.5%, and the corresponding displacement increased by 60.2% to 92.2%. This positive correlation arose because the three-dimensional fiber bridging network directly bore tensile stress, forming a dual enhancement mechanism with steel tube confinement, which transformed the material failure mode from brittle crushing to progressive failure. SFs significantly improved energy dissipation efficiency through the pull-out process, making them the optimal choice for simultaneously optimizing bearing capacity and ductility. At a dosage of 1.6%, the displacement increase rate could reach 92.2%.
(4) The core value of BFs lies in ductility optimization. The maximum improvement in the ultimate capacity of the specimens was only 7.4%, but the corresponding displacement increased steadily by 20.1% to 33.2%. This phenomenon was attributed to the low strength of the fibers themselves and the impact of their hydrophilicity on matrix compactness. However, fibers’ crack-bridging and progressive pull-out energy dissipation mechanisms significantly improved the fracture performance of concrete. This toughening effect had weak dependence on steel thickness, and a displacement increase of more than 20% could be achieved even when the steel thickness was 1.2 mm.
(5) Aiming to rectify the adaptability defect of Eurocode 4 to fiber-reinforced concrete, this study proposed a modified formula with a fiber correction factor *η*. The original formula, which ignored the fiber reinforcement mechanism and the transformation of failure modes, resulted in a prediction error of 6.3% for the bearing capacity of fiber-reinforced specimens. After modification, the calculation error of the formula was reduced to within 1%, with a standard deviation of only 0.016. This unified the fiber–steel–concrete synergistic working mechanism and provided a reliable theoretical basis for subsequent research.

The findings enable optimized fiber selection for prefabricated CFS-C structures. Subsequent research will investigate the enhancement mechanisms of these fibers under lateral impact loads to advance applications in impact-resistant structural systems.

## Figures and Tables

**Table 1:** Cross-sectional dimensions of composite columns.

Specimen Number	*T* (mm)	*D*	*h*_1_	*h*
L-1.2	1.2	140	50	90
L-2.0	2.0	140	50	90

**Table 2:** ABAQUS CDP parameter settings.

Parameter Name	Dilation Angle	Eccentricity	*f*_bo_ */f*_co_	*k*	Viscous Parameter
Set value	30	0.1	1.16	0.6667	0.0005

**Table 3:** Comparison of ultimate capacity of specimens.

Specimen Number	*P*_f_ (kN)	*P*_t_ (kN)	*P*_t_/*P*_f_
L-1.2-6	307.90	294.15	0.96
L-2.0-6	558.06	544.57	0.98
L-1.2-10	419.55	401.17	0.96
L-2.0-10	620.08	593.07	0.96

Note: *P*_f_ refers to the ultimate capacity of the axial compression finite element simulation specimen; *P*_t_ refers to the ultimate capacity of the axial compression test specimen.

**Table 4:** Basic characteristics of fiber materials.

Paper	Fiber Types	Diameter (mm)	Length (mm)	Tensile Strength (MPa)	Elastic Modulus (GPa)
Xu et al. [44]	PF	0.048	8	≥400	6.5
Zheng et al. [45]	CF	0.007	3	3450	230
Ni et al. [46]	SF	0.55	30	700–1100	206
Li et al. [47]	BF	0.3–0.4	30	≥520	≥24

**Table 5:** Comparison between calculated ultimate capacity and FEM values of specimens.

Specimen Number	*N*_u_	*P*_f_	*N*_u_/*P*_f_	Specimen Number	*N*_u_	*P*_f_	*N*_u_/*P*_f_
PF-1.2-0	700.48	695.75	1.007	PF-1.5-0	762.03	742.13	1.027
PF-1.2-0.5	728.11	656.09	1.110	PF-1.5-0.5	789.62	696.84	1.133
PF-1.2-1.0	717.47	681.02	1.054	PF-1.5-1.0	779.00	726.74	1.072
PF-1.2-1.5	712.19	675.35	1.055	PF-1.5-1.5	773.73	724.93	1.067
PF-1.2-2.0	679.82	637.96	1.066	PF-1.5-2.0	741.41	686.90	1.079
PF-1.8-0	820.96	768.09	1.069	PF-2.0-0	858.85	816.85	1.051
PF-1.8-0.5	848.50	744.14	1.140	PF-2.0-0.5	886.36	783.47	1.131
PF-1.8-1.0	837.89	795.12	1.054	PF-2.0-1.0	875.77	840.60	1.042
PF-1.8-1.5	832.63	781.35	1.066	PF-2.0-1.5	870.52	837.78	1.039
PF-1.8-2.0	800.40	746.70	1.072	PF-2.0-2.0	838.29	796.48	1.052
CF-1.2-0	684.47	687.95	0.995	CF-1.5-0	746.82	775.14	0.963
CF-1.2-0.2	704.68	734.61	0.959	CF-1.5-0.2	766.23	815.88	0.939
CF-1.2-0.4	745.93	784.76	0.951	CF-1.5-0.4	807.41	861.72	0.937
CF-1.2-0.6	623.03	635.68	0.980	CF-1.5-0.6	684.72	714.57	0.958
CF-1.2-0.8	587.34	576.73	1.018	CF-1.5-0.8	649.09	656.84	0.988
CF-1.8-0	805.00	853.77	0.943	CF-2.0-0	842.92	906.60	0.930
CF-1.8-0.2	825.15	892.97	0.924	CF-2.0-0.2	863.04	943.96	0.914
CF-1.8-0.4	866.25	939.02	0.923	CF-2.0-0.4	904.10	991.28	0.912
CF-1.8-0.6	743.77	784.69	0.948	CF-2.0-0.6	781.76	839.38	0.931
CF-1.8-0.8	708.21	731.18	0.969	CF-2.0-0.8	746.23	784.62	0.951
SF-1.2-0	699.93	696.66	1.005	SF-1.5-0	761.48	721.84	1.055
SF-1.2-1.0	737.28	790.06	0.933	SF-1.5-1.0	798.78	858.20	0.931
SF-1.2-1.3	771.93	855.26	0.903	SF-1.5-1.3	833.36	909.24	0.917
SF-1.2-1.6	796.15	866.76	0.919	SF-1.5-1.6	857.55	933.54	0.919
SF-1.8-0	820.41	731.67	1.121	SF-2.0-0	858.30	801.26	1.071
SF-1.8-1.0	857.64	917.70	0.935	SF-2.0-1.0	895.49	969.59	0.924
SF-1.8-1.3	892.16	933.26	0.956	SF-2.0-1.3	929.98	1015.91	0.915
SF-1.8-1.6	916.31	998.59	0.918	SF-2.0-1.6	954.09	1053.13	0.906
BF-1.2-0	748.61	779.31	0.961	BF-1.5-0	810.08	827.09	0.979
BF-1.2-0.25	735.02	780.46	0.942	BF-1.5-0.25	796.52	844.08	0.944
BF-1.2-0.5	708.98	762.07	0.930	BF-1.5-0.5	770.53	833.33	0.925
BF-1.2-0.75	698.79	758.62	0.921	BF-1.5-0.75	760.35	829.38	0.917
BF-1.8-0	868.92	846.63	1.026	BF-2.0-0	906.76	910.45	0.996
BF-1.8-0.25	855.38	908.89	0.941	BF-2.0-0.25	893.24	955.22	0.935
BF-1.8-0.5	829.43	889.64	0.932	BF-2.0-0.5	867.32	935.32	0.927
BF-1.8-0.75	819.28	883.98	0.927	BF-2.0-0.75	857.18	958.33	0.894

## Data Availability

Data will be made available on request.
